# Topographic effect on the phenology of *Ficus pedunculosa* var. *mearnsii* (Mearns fig) in its northern boundary distribution, Taiwan

**DOI:** 10.1038/s41598-017-14402-z

**Published:** 2017-11-07

**Authors:** Chu-Chia Kuo, Anthony Bain, Yu-Ting Chiu, Yi-Chiao Ho, Wen-Hsuan Chen, Lien-Siang Chou, Hsy-Yu Tzeng

**Affiliations:** 1 Department of Forestry, National Chung- Hsing University, Taichung, Taiwan; 2 Institute of Ecology and Evolutionary Biology, College of Life Sciences, National Taiwan University, Taipei, Taiwan

## Abstract

Mearns fig grows at the edge of coastal vegetation on uplifted coral reefs, its population and mutualistic-pollinators are susceptible to the influence of extreme weather. To determine the phenology of Mearns fig and the effects of various weather events under small-scale topographic differences, phenology was conducted for 3 years and 7 months. Results showed that Mearns figs had multiple leaf and fig productions year-round. Topographic effects caused population in Frog Rock Trail and Jialeshuei, which are less than 10 km away from each other, to exhibit different phenological patterns after experiencing severe weather events. Northeast monsoons led the Jialeshuei population to show low amounts of leaves and figs in winter and the phenological production was also susceptible to disturbances by typhoons in summer. Fig reproduction in such environment was disadvantageous to maintain pollinators. Besides, topographic complex in microhabitat of Frog Rock Trail protected some individuals from these same events thus safeguard population’s survival. The phenology of Mearns fig would respond to the weather events sensitively, which serve as references for estimating the mutualism system, and as indicators of climate change.

## Introduction

Plant phenology is a critical discipline of basic science that reveals the transformational mechanisms of plant life in various phases and plants relationships with the environment and climate^1^. Plants phenology not only affects the existence and reproduction of the plants themselves, but also other creatures that mainly inhabit these plants; feed on the leaves, flowers, or fruits of these plants; or helps pollinate the plants^2^. Currently, signs of climate change have become evident, rendering phenology an increasingly substantial component of global change studies. Specifically, increased frequencies of extreme climate events have changed plant phenologies^3^. If these climate changes intensify, the distribution borders of plant species will be affected, and existing terrestrial ecosystems will be altered^4,5^. A growing amount of modelling and empirical studies on plant phenology have indicated that phenological sensitivity can be adopted as an indicator of the long-term influence of climate change on creatures in terrestrial ecosystems^6–8^.

Approximately 750 species of plants in the *Ficus* genus exist worldwide, primarily in tropical-subtropical areas^9,10^. They exhibit various life-forms and provide highly nutritious fruits^11^ (called syconia) in stable amounts^12^ to provide food for many frugivore species. Therefore, figs serve as one of the keystone species in tropical forests^11–13^. In addition, *Ficus* are characterized by a peculiar fruiting phenology and mutualism with their specific pollinating wasps. Fig phenology and life history of pollinators are closely interconnected^14,15^. Because of the special mutualistic relationship and the importance of figs in tropical ecosystems, fig phenology continues to be a focus of research^16,17^.

Figs are the venues for *Ficus* to produce seeds and grow pollinators. Thus, the specific fig phenology is the mechanism ensuring the survival of pollinator^18^. Accordingly, phenological patterns were established on the basis of the long-term interaction on the coevolutionary relationship between *Ficus* and pollinators under stable climatic conditions^19–21^. Previous studies on *Ficus* phenology have mainly investigated the correlation between climate factors and the fig phenology^18,22–27^. Temperature and rainfall are key climatic factors that often are correlated with the fig phenology^24,25,27–29^. In regions with clear seasonality, season changes serve as indicator for phenological events, for example the beginning of the wet season initiates leaf flushing and fig production^22,23^. However, few studies have explored the effects that extreme weather events^30^ or topography^31^ have on *Ficus* phenology. For example, Harrison noted that a severe drought induced by El Niño in northern Borneo in 1998 caused the regional extinction of the pollinators of many fig species^30^. In addition, the phenological variations of a single *Ficus* species in different locations (i.e. environments) have been the topic of only few studies^18,21,31^.

*F. pedunculosa* var. *mearnsii* (Mearns fig) inhabits only on coastal uplifted coral reefs which is considered as a harsh environment with thin soil, scarce nutrition and fresh water coupled frequent sea-sprays increasing the environment salinity. The Mearns fig is classified as a vulnerable species^32^. Taiwan is the northern boundary of Mearns fig distribution, where a few populations are distributed in a narrow coastal area on the South of Taiwan. Recently the consequences of climate change, such as the raising sea level has affected many coastal ecosystems^33,34^ and the increased frequency of strong typhoons in Taiwan^35^ threatens the Mearns fig populations.

**Figure 1:**
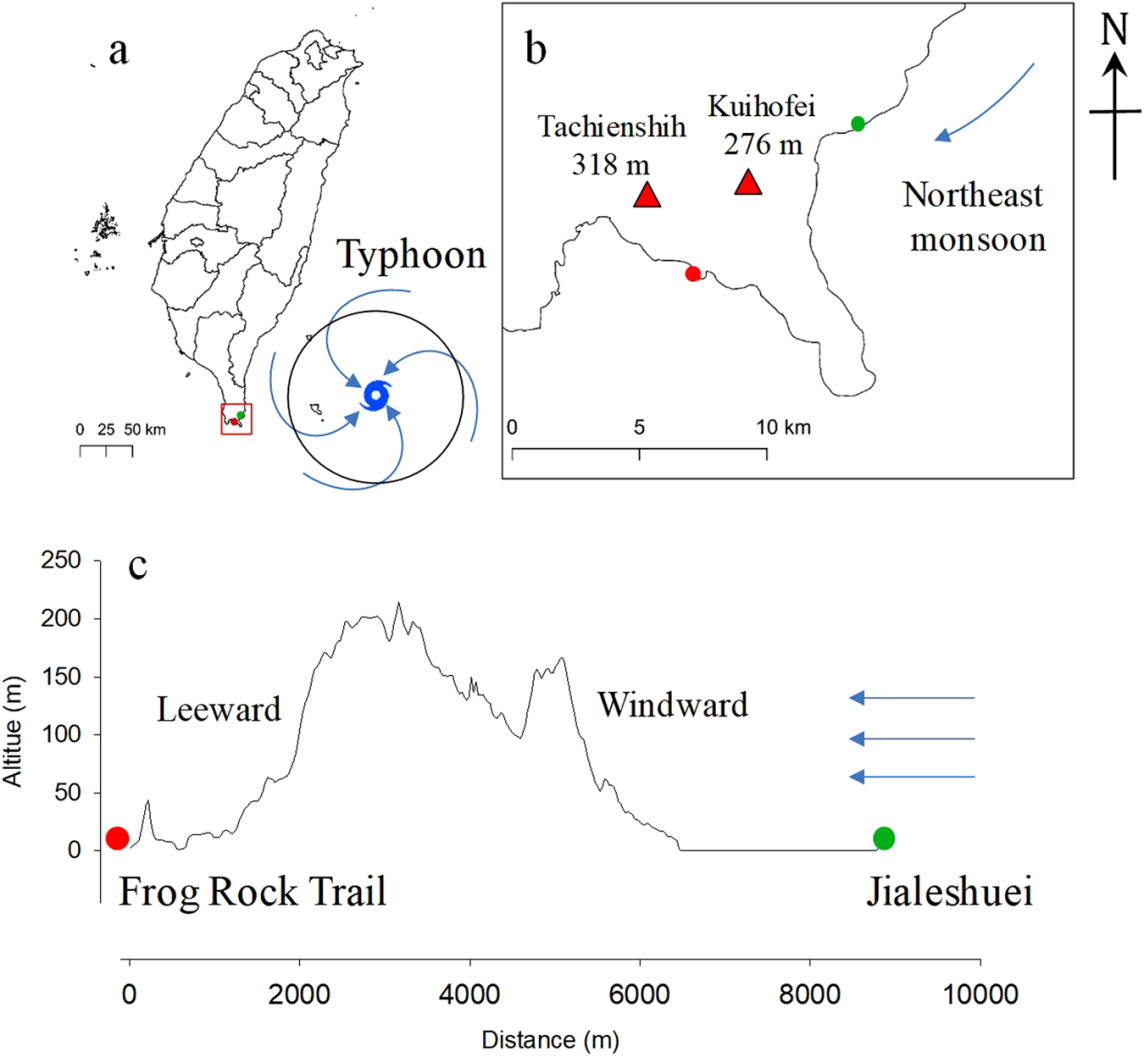
Illustrations of the location, topographic characteristics, and weather event effects of the study sites. (**a**) Effect of typhoons in the summer. (**b**) Effect of northeast monsoons in the winter. (**c**) Topographic effects; the red doc indicates Frog Rock Trail, the green dot indicates Jialeshuei, and the blue arrows indicate wind direction, map were generated with QGIS (version 2.18, Open Source Geospatial Foundation Project, Boston, USA, www.qgis.org).

The south of Taiwan is marked by clear seasonal changes, with typhoons and southwestern airflows in the summer, and northeastern monsoons in the winter. Nevertheless, the topography of this area (Fig. 1) generates considerable climate differences that can affect the species composition, structure, and physiognomy of its forest^36^. However, few studies have investigated whether such small-scaled climatic differences derived from topography can influence the phenology of plants. This is crucial information for fig species which rely on fig phenology to ensure the survival of the pollinator populations. Thus, the present study monitored the phenology of two Mearns fig populations in different habitats for more than three years and half. The aim of the study is to investigate if differences exist in the fig phenology of the Mearns fig living under different environmental conditions induced by topography, as well as the phenological reaction of Mearns fig under extreme climatic events.

## Results

**Figure 2:**
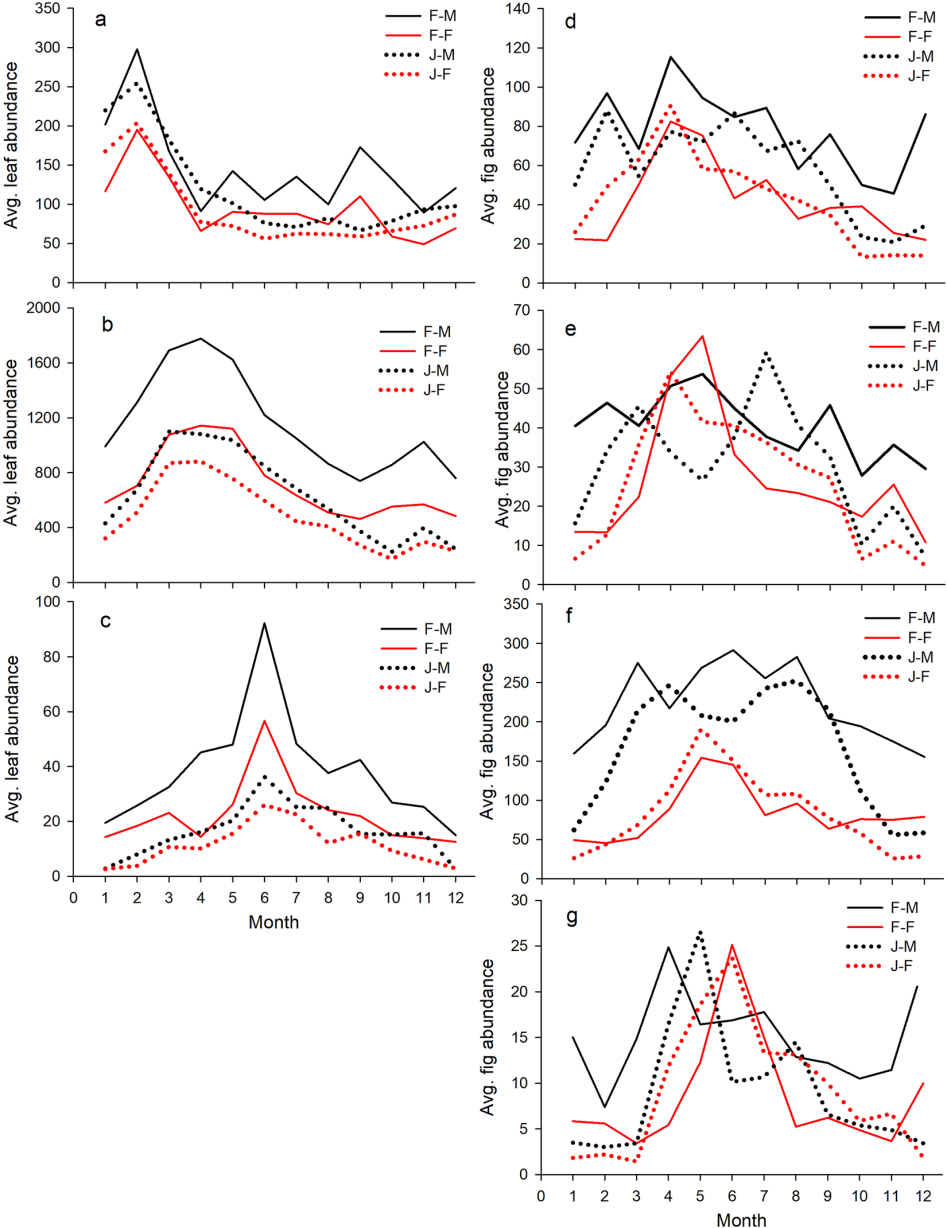
Variations in the average monthly total abundance of leaves and figs in various development phases. F-M: Frog Rock-Male, F-F: Frog Rock-Female, J-M: Jialeshuei-Male, J-F: Jialeshuei-Female, (**a**) Tender leaf, (**b**) Mature leaf, (**c**) Senescent leaf, (**d**) A phase, (**e**) B phase, (**f**) C phase, and (**g**) D & E phase.

**Figure 3:**
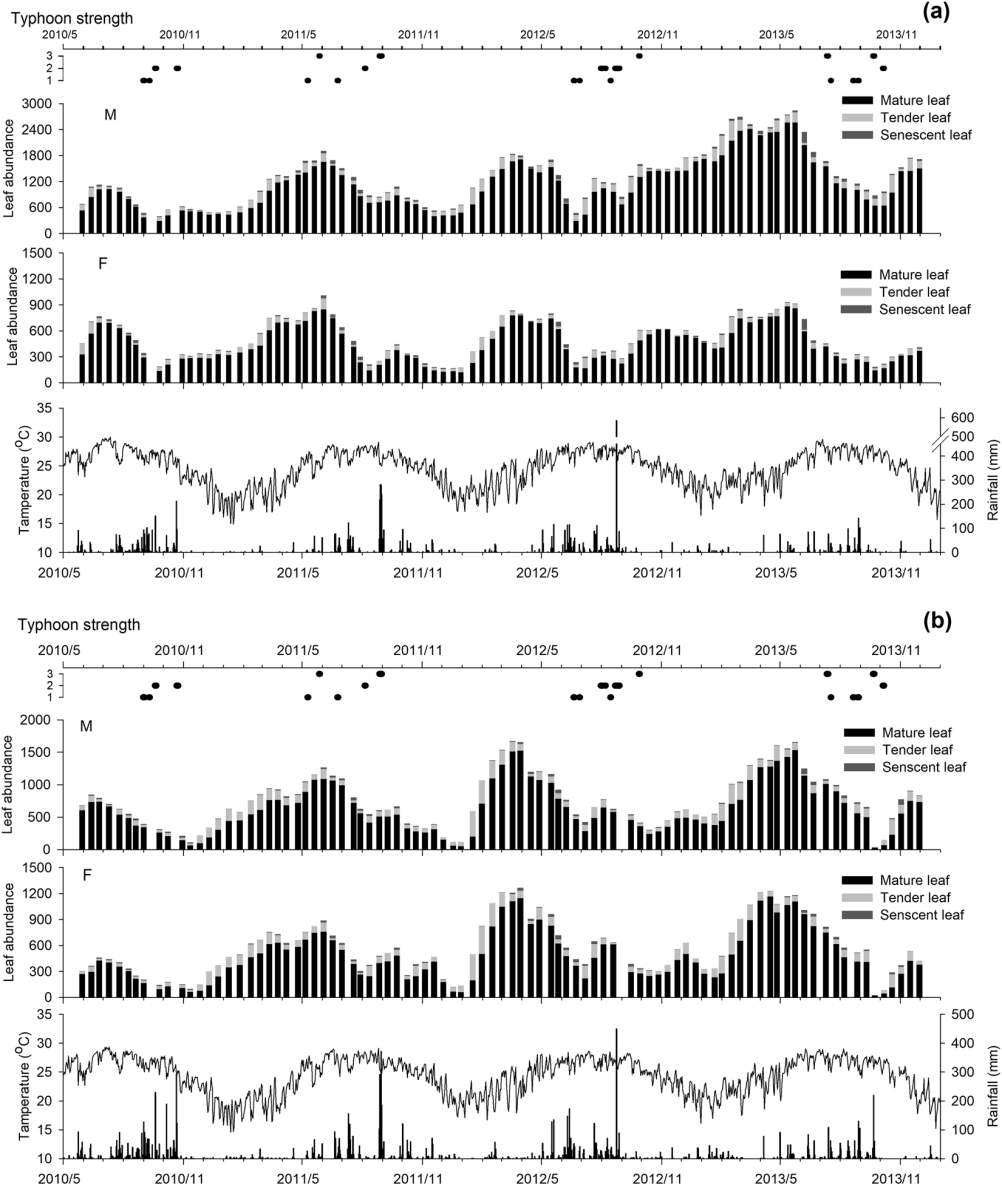
Variation of total leaf abundance at the Frog Rock Trail (**a**) and at Jialeshuei (**b**). M: Male, F: Female.

**Figure 4:**
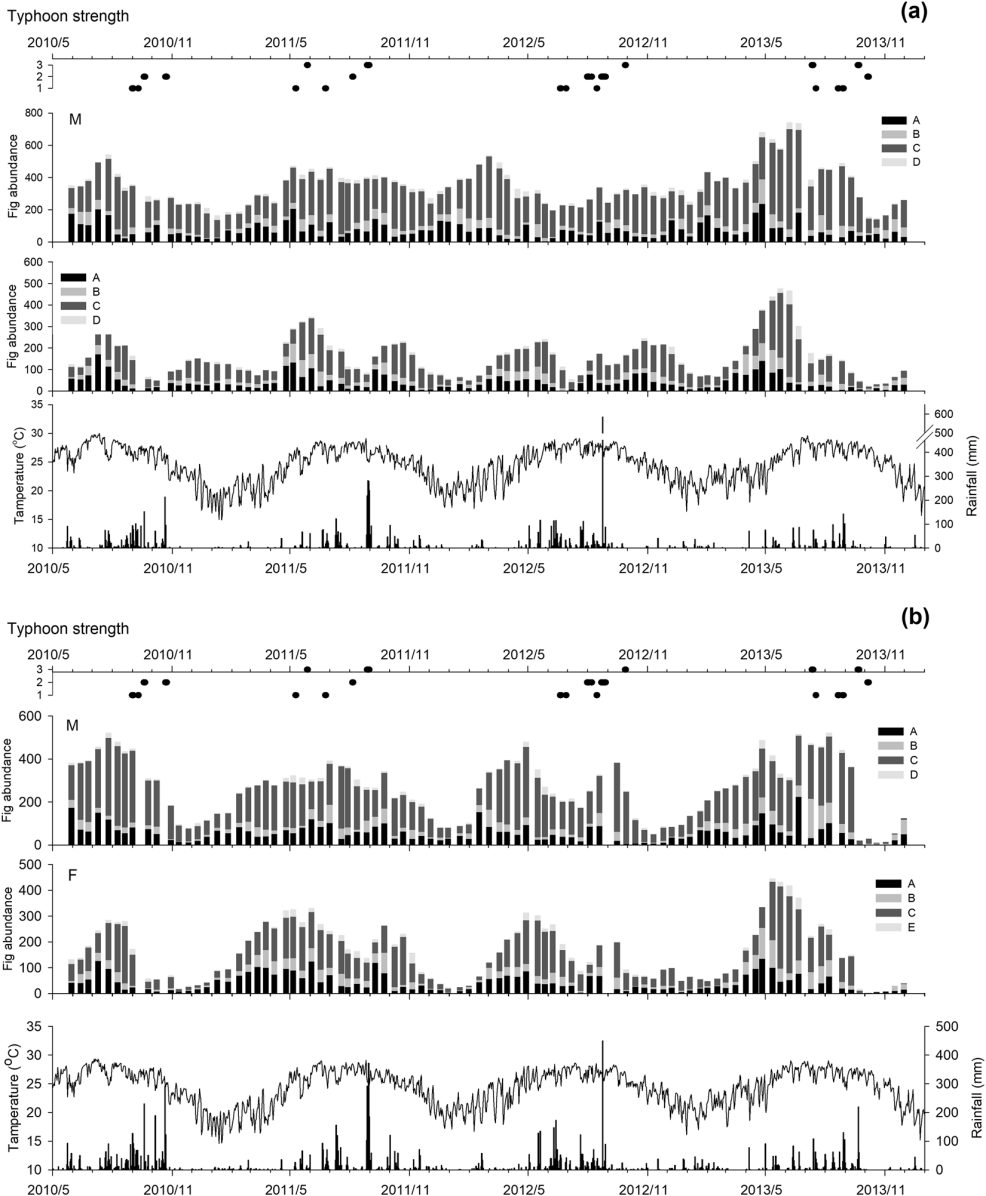
Variation of total fig abundance at the Frog Rock Trail (**a**) and at Jialeshuei (**b**). M: Male, F: Female.

The research sites are located in a tropical area with clear seasonal differences, the leaf replacement displayed a semi-deciduous pattern and the fig production varied seasonally (Figs 2–4). Regardless of sex or population, the leaf and fig phenologies exhibited a significant time auto-correction (Supplementary Table S1). However, variations in Mearns fig phenology were not completely influenced by temperature and rainfall; the occurrence of northeast monsoons in the winter and typhoons in the summer-autumn both also affected Mearns fig phenology.

### Leaf phenology

The seasonal pattern of leaf abscission and sprouting observed in both males and females Mearns figs in the Frog Rock Trail and Jialeshuei habitats exhibited consistent trends (Fig. 2). The abundance of leaf at different developmental phases showed high positive significantly correlations between them (Supplementary Table S2), suggesting no sexual difference in leaf phenology. Moreover, variations in tender leaf abundance indicated that both males and females in the two sites produced tender leaves year-round, with the period between January and March marking the peak of leaf sprouting (Fig. 2). Along with the gradual maturation of tender leaves, the abundance of mature leaves peaked from March to June. Subsequently, the abundance of senescent leaves peaked in June and July. The period between October and January was a low abundance period for mature leaves.

Furthermore, leaf phenology variations by years explored according to total leaf abundance variations from 2010 to 2013 (Fig. 3); particularly in 2012, a unique phenomenon identified. Among the Frog Rock Trail population, the lowest value of leaf abundance occurred in August (Fig. 3a), and it gradually increased afterwards to reach its peak the following May (2013). Meanwhile, changes in the total leaf abundance of the Jialeshuei population occurred from November to February of the following years (Fig. 3b). Overall, Mearns fig in Frog Rock showed smaller degrees of leaf abundance variation throughout the observed period compared with the population in Jialeshuei. The periods in which the two sites displayed greater differences in time scale mainly comprised the typhoon season (June–August) and the northeastern monsoon season (October–April).

### Fig phenology

This study also revealed that the males and females of both Mearns fig populations produced figs throughout the year to maintain a relative stable level of fig abundance (Fig. 2). Variations in the monthly average of total fig abundance adopted to represent the basic production and development trends of figs, and the results showed fig production trends differed between males and females. In addition, the fig production trends of plants within gender but from different sites mostly exhibited similarities, with some locational variations.

Of all the development phases, Phase C figs require the longest development time; hence, the fig abundance at this phase was higher than that at other phases (Fig. 2). In Frog Rock Trail, male Phase C figs had two peak of abundance in March and June-August; the lowest of abundance occurred from November to January the following year. By contrast, the amount of Phase C fig produced by females was lower; the single abundance peak was between May and June, and the fig quantity was relatively low in other periods. In Jialeshuei, males also had two peaks of Phase C fig production, May-April and July-September; meanwhile, females reached their peak of Phase C fig abundance in May and produced relatively few at other times. Comparison with the male Phase C fig production trends between Jialeshuei and Frog Rock Trail, the males of Frog Rock Trail reached peak abundance of Phase C fig one month earlier and maintained at a certain amount of Phase C fig in December. The production trends of female Phase C fig of two sites were similar.

A comparison of the fig abundance variations by year (Fig. 4) revealed that for male Mearns fig in Frog Rock Trail, the fig abundance maintained at a relatively stable level throughout the study period (Fig. 4a). Considerably small amount of fig appeared only in July and October of 2013. However, males in Jialeshuei showed greater variations in fig abundance. Notably lower levels of fig abundance occurred in November 2010-January 2011, January-March 2012, October- December 2012, and October-November 2013. Occasionally, only Phase C figs remained, which lasted for a relatively long time (Fig. 4b). Specifically, females in Frog Rock Trail had two notable reproduction cycles per year; those in Jialeshuei also had two reproduction crops, however, due to the plants exhibited a considerable decrease in fig abundance during the winter, the second was not distinct.

### Relationship between phenology and climate

Tender leaves indicate the beginning of leaf life-cycle, and matured leaves are crucial to synthesize nutrition and support plant growth. Similarly, Phase A figs indicate the initial stage of fig life-cycle, and Phase C fig is essential to feed wasps and produce seeds. Moreover, these two phases are the longest development periods of a fig. Therefore, a correlation analysis was performed on the Phase A and Phase C fig abundances, tender and mature leaf abundances, and temperature and rainfall (Tables 1 and 2).

**Table 1:** Analysis of the correlation between temperature, rainfall, and the various phases of leaf and fig phenology of the *F. pedunculosa* var. *mearnsii* in Frog Rock Trail.

Fig phase		coefficient	Male		Female		Temperature	Rainfall
Leaf phase			A	C	A	C		
Male	Tender leaf	rho	**0.541**	**−0.246**			**−**0.135	**−**0.051
		p	0.000	0.019			0.209	0.635
		N	90	90			89	89
	Mature leaf	rho	0.041	**0.465**			**−**0.101	**−**0.181
		p	0.700	<0.001			0.346	0.089
		N	90	90			89	89
Female	Tender leaf	rho			**−**0.010	**−0.376**	**−**0.120	**−**0.101
		p			0.922	<0.001	0.265	0.345
		N			90	90	89	89
	Mature leaf	rho			**0.633**	**0.338**	**−**0.122	**−**0.197
		p			<0.001	0.001	0.256	0.064
		N			90	90	89	89
Temperature		rho	**−**0.063	**0.342**	0.047	**0.242**		
		p	0.559	0.001	0.664	0.023		
		N	89	89	89	89		
Rainfall		rho	**−**0.023	0.148	**−**0.074	**0.214**		
		p	0.828	0.165	0.492	0.044		
		N	89	89	89	89		

Bold letter: Significance = 0.05 (two-tailed).

**Table 2:** Analysis of the Spearman’s rank correlation between temperature, rainfall, and the various phases of leaf and fig phenology of the *F. pedunculosa* var. *mearnsii* in Jialeshuei.

Fig phase		coefficient	Male		Female		Temperature	Rainfall
Leaf phase			A	C	A	C		
Male	Tender leaf	rho	0.134	**−0.236**			**−0.538**	**−0.225**
		p	0.21	0.026			< 0.001	0.034
		N	89	89			89	89
	Mature leaf	rho	**0.517**	**0.494**			0.037	0.141
		p	<0.001	<0.001			0.729	0.186
		N	89	89			89	89
Female	Tender leaf	rho			0.114	**−0.23**	**−0.49**	**−0.242**
		p			0.286	0.03	<0.001	0.022
		N			89	89	89	89
	Mature leaf	rho			**0.719**	**0.485**	**−**0.032	0.115
		p			<0.001	<0.001	0.764	0.283
		N			89	89	89	89
Temperature		rho	**−**0.063	0.125	**0.498**	0.014		
		p	0.559	0.242	0.000	0.897		
		N	89	89	89	89		
Rainfall		rho	**−**0.023	0.194	**0.309**	0.079		
		p	0.828	0.069	0.003	0.459		
		N	89	89	89	89		

Bold letter: Significance level = 0.05 (two-tailed).

For male Mearns fig in Frog Rock Trail, Phase A fig was positive correlated significant with tender leaf abundance (Table 1). Phase C fig was negative correlated obviously with tender leaf abundance, but positive correlated with mature leaf abundance significantly. For females, Phase A fig was positive correlated with mature leaf abundance significantly. Additionally, Phase C fig was negative correlated significantly with tender leaf abundance, but positive correlated with mature leaf abundance significantly. For both genders, their mature leaf abundance negative correlated with rainfall significantly. Moreover, only Phase C fig of both genders correlated significant with temperature positively.

For male Mearns fig in Jialeshuei (Table 2), Phase A fig was positive correlated with mature leaf abundance significantly. Additionally, Phase C fig was negative correlated significant with tender leaf abundance, but positive correlated with mature leaf abundance significantly. For females, Phase A fig was positive correlated significant with mature leaf abundance. In addition, Phase C fig was negative correlated significant with tender leaf abundance, but positive correlated with mature leaf abundance significantly. Both males’ and females’ mature leaf abundance were positive correlated with temperature and rainfall significantly. Moreover, Phase C figs of both genders were positive correlated with temperature and rainfall significantly, but Phase A figs did not.

Weather events, such as typhoons, serve as other critical influencing factors on Mearns fig phenology. Notably, not only leaf but also fig abundances of both populations showed markedly decreased during the typhoon seasons, namely September to October in 2010, June to October in 2012, and July to October in 2013 (Figs 3 and 4). On August 22, 2012, the high tide accompanying Typhoon Tembin destroyed several parts of the coral reef at Jialeshuei, caused six of sampled trees to disappear, and damaged the aboveground of all plants almost. On September 21, 2013, Typhoon Usagi devastated Southern Taiwan with another high tide, some coral reefs at Jialeshuei was broke, triggering the complete loss of five sampled trees and withering the aboveground portions of another 16 trees. After Typhoon Usagi, only 20 male Phase C figs remained in the study area. The typhoon also caused numerous samples in Frog Rock Trail to lose all of their leaves and figs. However, the most serious event was Typhoon Fitow in October 2013, which caused the fig abundance in Frog Rock Trail to reach the lowest quantity, and completely damaged the Phase C figs that remained on male trees in Jialeshuei. In addition, during the north-eastern monsoons from October to March, the leaves of Mearns figs in Jialeshuei substantially withered; fig abundance also decreased (Fig. 4).

## Discussion

Plants reveal their sensitivity to environmental variations through phenological performances^37^. The two populations of Mearns fig examined were located in habitats with merely 9.2 km of distance between them, and exhibited both similar and somewhat different phenological patterns. The phenology similarities generally derived from the biological characteristics of the species itself, whereas the phenological differences were due to the effects of climates on location and topographic differences of these two habitats. Hence, the two populations differed in leaf and fig productions under identical weather events when examined on a large temporal and spatial scale. These differences in phenology demonstrated that Mearns fig can adapt flexibly to dissimilar environmental conditions, in particular to harsh habitats.

The leaf phenologies of male and female Mearns fig trees in Frog Rock Trail and Jialeshuei were similar, suggesting that plant variations mainly triggered by climate^10,20,23,26,27^ and not closely related to sexual distinction. In our analysis of leaf phenology and meteorological factors, we found that the mature leaf abundance of Mearns fig in Frog Rock Trail negative correlated with rainfall significantly, whereas in Jialeshuei, the mature leaf abundance negatively correlated with both rainfall and temperature. However, the tender leaf abundance of the two populations did not correlate significantly with either temperature or rainfall. Instead, tender leaves and Phase A figs were produced almost continuously throughout the year, indicating that temperature and rainfall would not the main factors influencing the reproduction. However, in tropical or dry areas, rainfall cycle is a key component of plant reproduction^38^. Indeed, several studies on the phenology of *Ficus* have found that the abundance of tender leaves, mature leaves, and Phase A figs were correlated positively with rainfall^18,23,25,26^. Although the results of the present study differ from those of previous scholars, this difference does not imply the negative influence that water exerts on Mearns fig phenology. Specifically, the substantial decrease in mature leave abundance associated with rainfall mostly attributed to the physical damage induced by typhoons and south-western airflows with severe heavy precipitation and strong winds. In addition, we found that in dry environments, scant rain is sufficient to meet the growth requirements of Mearns fig and enable them to unfold leaves or produce figs (Figs 3 and 4); this verifies the excellent adaptation abilities of Mearns fig to dry environments.

Clear sexual differences in fig production appeared in Mearns fig in both sites. The males exhibited multiple production cycles, which indicates that the abundance of male Phase C figs (wherein wasp larvae or pupas can feed) maintained at a relatively stable level. Thus, the phenology of male figs related to the survival of pollinator population. Adjustments in the fig developmental cycle of male trees can enhance the fitness of pollinators, and thereby facilitate the population expansion of *Ficus* ^14,38,39^. By comparison, female Mearns fig have only one or two fig abundances within year, a clear seasonal characteristic, which suggests that females produce seeds in the most appropriate season to facilitate *Ficus* reproduction^38^.

Dioecious *Ficus* distributed typically in clusters^40,41^. Moreover, their pollinating wasps often come from the surrounding vicinity, i.e., places within a short accessible flying distance. The size pollinator population is dependent on male fig abundance. Therefore, groups of these plants are typically highly dense in a growth site, and the male trees have frequent fig production cycles; because the cycles are highly unsynchronised between the plants, the abundance of pollinating wasps can be maintained at a specific level^10,24,40,42–44^. Mearns figs are distributed in this typical distribution pattern of dioecious *Ficus*, and in the present study, clear differences were identified between the males and females regarding fig abundance.

Leaf unfolding and reproduction occur that also serve as the plants’ carbon sink, and the manifestations of the phenological cycles of these two processes correlate with each other^27,45^. These correlations could be the reason for both genders that tender leaf abundances negatively correlated with Phase C fig abundances, and that Phase C fig abundances positively correlated with mature leaf abundances. Because Phase C figs denote the period when the seeds and wasp larvae develop inside it (i.e., the nutrition-providing phase) resulting in a strong carbon sink. When a considerable amount of Phase C figs is produced, numerous mature leaves are required to serve as a rich carbon source for the plant. Therefore, mature leaf abundances positive correlate with Phase C fig abundance significantly. Conversely, tender leaves also serve as a carbon sink of the plant. This is crucial because under the limited nutrition supply from mature leaves, maintaining a substantial number of Phase C figs at the same time would certainly cause severe competition in the nutrition supply. Thus, when supplying nutrients to facilitate the growth tender leaves, Mearns fig lose mature leaves and become incapable of sustaining an abundant of Phase C figs.

The effects of interference vary on a micro-scale according to heterogeneous topography. Hence, the same interference events can exert diverse effects on different plant populati7ons^36,46^. Although the spatial distance between Frog Rock Trail and Jialeshuei was short, phenological differences occurred in the populations at these two sites. Such differences primarily derive from the effects of geological location, topographic characteristics, the micro-environmental differences in plant habitat, and the interactions between these topographic effects and climate. Thus, the two Mearns fig populations face different microclimate events, such as precipitation, foehn, and salt spray, during identical climate events (i.e., typhoons and monsoons), and thereby present varying phenological patterns.

Frog Rock Trail is located at the southern end of Hengchun Peninsula and Jialeshuei is located at the east side. Their topographic locations cause Frog Rock Trail and Jialeshuei to be at the leeward and windward sides of typhoons and north-eastern monsoons, respectively (Fig. 1). Because of the northeast-monsoon-induced salt spray and strong wind that occurs in winter, Mearns figs on the east shore in Jialeshuei exhibited considerably decreased leaf and fig abundances in winter every year (Fig. 3). The phenomenon is similar to the leaf withering of *F. septica* on the northeastern cape of Taiwan that also occurs because of the salt spray brought by the northeastern monsoon in winter^47^. By contrast, Frog Rock Trail is shaded by 200–300 m foothills, such as Kuihofei and Tachienshih (Fig. 1c); therefore, it less influenced by northeast monsoons, and Mearns figs maintained leaf and fig abundances at set levels in winter (Figs 3 and 4). Moreover, a dry northeast monsoon can aggravate the damage of the Jialeshuei Mearns figs, but a humid northeast monsoon can supplement the water supply of this upwind population.

Typhoons is a key climate factor in Taiwan during summer-autumn that affect the structure and composition of plant communities^48^. A total of 193 typhoons struck Taiwan from 1958 to 2015, at an average of 3–4 typhoons per year. Although the frequency of typhoon strikes is high in Taiwan, typhoons can cause various effects based on their path, intensity, and retention time. Compared with the Mearns figs in Jialeshuei, the population in Frog Rock Trail was relatively capable of maintaining its leaf and fig abundances at specific levels after severe weather events. This phenomenon also attributed to the diversity in the microhabitat of Frog Rock Trail, which provided Mearns figs with more appropriate shelter during such events. By contrast, the vegetation in the microhabitat of Jialeshuei was mainly composed of Mearns fig; the damage caused by high-intensity special weather events was also greater. Nevertheless, the leaf and fig abundances of Mearns fig in both Frog Rock Trail and Jialeshuei gradually recovered from typhoon strikes and successfully re-reached their peaks in May or June of the following year. This phenomenon might reflect an adaptation of Mearns fig that inhabits on uplifted coral reefs with high frequency disturbed by typhoons^18^.

However, due to the effect of drastic weather events, would cause nearly all leaves and figs of all trees in a region to renew together, and leads to the local disappearance or even extinction of pollinator local-population^30,40^. During the observation period, the aboveground portion of Mearns fig in the two habitats were found to rapidly recover after weather events such as typhoons by regrowing branches, leaves, and figs. Then, in 2013, Typhoon Usagi struck the areas towards the end of the autumn, followed by a northeast monsoon that occurred that winter. These dual events caused fig abundance of Jialeshuei Mearns figs to decline into a very low quantity; additionally, the Phase C male figs, which are critical to the survival of pollinators, nearly vanished (Fig. 4b). Because dioecious *Ficus* depend on unsynchronized between- fig production cycles to enhance the fitness of their pollinator population, the synchronization of the reproduction cycle induced by special weather events reduces the survival and reproduction rates of the pollinators^21,47^. Overall, although Mearns figs exhibit excellent tolerance to uplifted coral reef, excessively intense weather events can cause irreparable damage to the plants and affect the survival of pollinators.

Species living in an ecotone are susceptible to the influence of neighboring ecosystems. When disturbances occur and lead to changes in the ecotone, population abundance of the species in the ecotone often declines^46,49^. Mearns figs are limited to growing in an ecotone bordered by the coastal vegetation and marine ecosystems, where substantial environmental variations occur. In addition to the competition from other plants, Mearns figs can be damaged physically by tides or wind, which can also affect their phenological cycles and population abundance. The results of this study indicated that Mearns figs were flexible in their phenological cycles according to the microclimate differences caused by topographic effects. This verifies the adaptation and survival strategy of this species in a severe coastal uplifted coral reef environment. Nevertheless, the resistance of Mearns figs to environment variations is limited. For example, although the plants can survive under high-intensity interference, local extinction of the exclusive pollinators of this species may occur.

Taiwan is the northern boundary of Mearns fig distribution, and is thus located on the margins of its geographical distribution. The geographical distribution boundary of a species would indicate its range of physical tolerance. Whether populations on the margins expand or shrink depends on individual adaptability to the environment^29,50,51^. Therefore, when environmental pressure escalates, the distribution range of a species may be reduced because of an inability to adapt^16^. Notably, the distribution boundary of Mearns fig has been threatened in recent years due to the influence of global climate change, specifically because of rising sea levels and the increasing frequency of extreme weather events^3^. If the occurrence of drastic events such as Typhoon Usagi increases, then the survival of Mearns fig in Taiwan will threatened. We suggest that further research on Mearns fig should be conducted, and that *ex situ* conservation should be performed on existing population to maintain the abundance of pollinating wasp population.

## Materials and Methods

### Study species

*Ficus pedunculosa* Miq. var. *mearnsii* (Merr.) Corner is a shrub, dioecious fig belonging to *Frutiscentiae* subsection of the *Ficus* subgenus^15,43^, which is distributed on uplifted coral reefs of Luzon Island of the Philippines, southern Taiwan, Green Island, and Orchid Island^9,15,44^. Taiwan is the species northern-boundary distribution^7,14^. Its associated pollinating wasp species is *Blastophaga pedunculosae* ^52^.

### Research Sites

Two Mearns fig habitats, Frog Rock Trail (21°56′40.2″N, 120°46′58.9″E) and Jialeshuei (21°59′39.6″N, 120°51′50.9″E) in the Kenting National Park (Fig. 1) in the tropical zone, were selected as study sites. They are located in the Hengchun Peninsula. The straight distance between these two areas is 9.2 km. All of the monitored Mearns figs live on uplifted coral reefs 2–10 m above sea level.

Hengchun Peninsula features a tropical dry climate^53^. According to 2010–2013 meteorological data (Central Weather Bureau of Taiwan), the annual average temperature at the Frog Rock Trail was 24.4 °C, ranging from 20.2 °C (January) to 27.7 °C (July), and at Jialeshuei 24.1 °C ranging from 19.7 °C (January) to 27.3 °C (July). The average annual rainfall at the Frog Rock Trail was 2576.0 mm (range: 37.1 in January–763.0 in August), and 3068.1 mm at Jialeshuei (range: 37.1 in January–807.6 in August).

### Phenological census

Seventy trees (39 males and 31 females) were selected from Frog Rock Trail, whereas 50 individuals (25 males and 25 females) were sampled from Jialeshuei. On each samples, 5–10 branches about 10–30 cm long were marked. Due to the biotic factor (branches self-thinning, effect by pests and diseases, etc.) and abiotic (typhoons, monsoon and drought etc.) factors, some sampled branches have died during the phenological survey period, so new branches of the same tree have been added to the phenological survey in order to maintain the number of monitored branches. The relationships between new and total sampled branches of fig and leaf abundances had shown in Supplementary Figures S1 and S2. The phenological survey was carried out from May 2010 to December 2013 at intervals of 10–15 days (average: 14.2 ± 2.2 days). In total, 90 and 89 surveys were conducted at Frog Rock Trail and Jialeshuei, respectively. In each survey, the amounts of leaves and figs in various development phases on each branch were documented; the leaves were categorized into tender, mature, and senescent leaves accordingly^25^. Fig development of the Mearns fig comprises five phases as following: pre-female phase (phase A), female phase (phase B), interfloral phase (phase C), male phase (male phase D), and ripe phase (female phase E). Male fig development does not have a phase E, and female fig development does not have a phase D^54^.

### Topographical and meteorological data

We estimated the map and topographic profile (Fig. 1) using 20 m digital elevation model (DEM) and cities map of Taiwan from Open Platform for Government Information of Taiwan (Open source database, http://data.gov.tw/).

Temperature and rainfall data were collected from the Kenting and Jialeshuei Automatic Weather Stations, which were established by the Central Weather Bureau of Taiwan (CWB) and measure both temperature and rainfall daily. Typhoon data were retrieved from a CWB database. A list of all typhoons that formed during the research period that had been issued with a warning were compiled, and the typhoons were classified into Grade 1 (mild), Grade 2 (moderate), or Grade 3 (strong) according to the CWB typhoon classification system.

### Data analysis

The map and topographic profile were generated with QGIS (version 2.18, Open Source Geospatial Foundation Project, Boston, USA, www.qgis.org).

Figure plotting and analysis were thus conducted on the total amount of growth to display the phenological variations of populations. The monthly averages of leaf and fig abundances at different developmental phases were calculated in order to show the yearly trends. Differences in leaf phenology from the two habitats were also explored. Subsequently, leaf and fig abundances at different developmental phases were summed and averaged by month to calculate the average total abundances of leaves and figs per phase in each month. Finally, the monthly average total abundances per year were calculated.

Phenology-related ecological data often exhibit autocorrelation in a time series^55^; hence, an autocorrelation function (ACF) was used to examine autocorrelation in the times series of leaf and fig abundance. Q statistics were adopted to examine the correlation coefficients, and gretl2017a statistical software was employed for the ACF analysis. Because phenological data was not normally distributed and our sample size was unequal between sites, the Spearman’s rank correlation was carried out using SPSS20 to analyse whether total abundance variations were correlated with climate trends.

### Data availability

The datasets generated during and/or analysed during the current study are available from the corresponding author on reasonable request.

## Electronic supplementary material

Supplementary Information
