# Supplementary material for: Topographic effect on the phenology of Ficus pedunculosa var. mearnsii (Mearns fig) in its northern boundary distribution, Taiwan
Source: Sci Rep. 2017 Nov 7;7:14699. doi: 10.1038/s41598-017-14402-z (PMC5676713; doi:10.1038/s41598-017-14402-z)
Supplement: Supplementary file 1 — Supplementary Information [file 41598_2017_14402_MOESM1_ESM.pdf]

Topographic effect on the phenology of *Ficus pedunculosa* var. *mearnsii* (Mearns fig) in its northern boundary distribution, Taiwan

Chu-Chia Kuo[1], Anthony Bain[1, 2], Yu-Ting Chiu[1],
Yi-Chiao Ho[1], Wen-Hsuan Chen[1], Lien-Siang Chou[2], Hsy-Yu Tzeng[1,*]

[1] Department of Forestry, National Chung- Hsing University, Taichung, Taiwan.

[2] Institute of Ecology and Evolutionary Biology, College of Life Sciences, National Taiwan University, Taipei, Taiwan.

[*] Corresponding author. E-mail: erecta@nchu.edu.tw

Supplementary

Table S1. Time autocorrelation analysis of leaf and fig phenology in Frog Rock Trail and Jialeshuei.

Study area		LAG	Male			Female		
			ACF	Q-stat.	p-value	ACF	Q-stat.	p-value
Frog Rock	Leaf	1	0.932	80.821	<0.001	0.899	75.234	<0.001
		2	0.820	144.051	<0.001	0.731	125.478	<0.001
		3	0.705	191.316	<0.001	0.569	156.292	<0.001
	Fig	1	0.817	62.112	<0.001	0.859	68.711	<0.001
		2	0.626	98.935	<0.001	0.586	101.017	<0.001
		3	0.503	122.976	<0.001	0.282	108.591	<0.001
Jialeshuei	Leaf	1	0.905	75.387	<0.001	0.885	72.052	<0.001
		2	0.734	125.532	<0.001	0.712	119.251	<0.001
		3	0.543	153.295	<0.001	0.521	144.851	<0.001
	Fig	1	0.846	65.877	<0.001	0.881	71.432	<0.001
		2	0.648	104.919	<0.001	0.709	118.172	<0.001
		3	0.419	121.446	<0.001	0.498	141.514	<0.001

Table S2. Spearman's rank correlation analysis of the various phases of leaf phenology exhibited by male and female *F. pedunculosa* var. *mearnsii* plants in Frog Rock Trail and Jialeshuei.

Sexual	Study area	phase	coefficient	Male		
				TL	ML	SL
Female	Frog Rock	TL	rho	**0.885**	0.086	-0.149
			p	<0.001	0.418	0.162
			n	90	90	90
		ML	rho	-0.025	**0.921**	**0.256**
			p	0.815	<0.001	0.015
			n	90	90	90
		SL	rho	-0.198	**0.229**	**0.795**
			p	0.062	0.030	<0.001
			n	90	90	90
	Jialeshuei	TL	rho	0.163	**0.886**	**-0.314**
			p	0.126	0.000	0.003
			n	89	89	89
		ML	rho	**0.933**	**0.313**	**0.236**
			p	<0.001	0.003	0.026
			n	89	89	89
		SL	rho	**0.266**	**-0.287**	**0.781**
			p	0.012	0.006	<0.001
			n	89	89	89

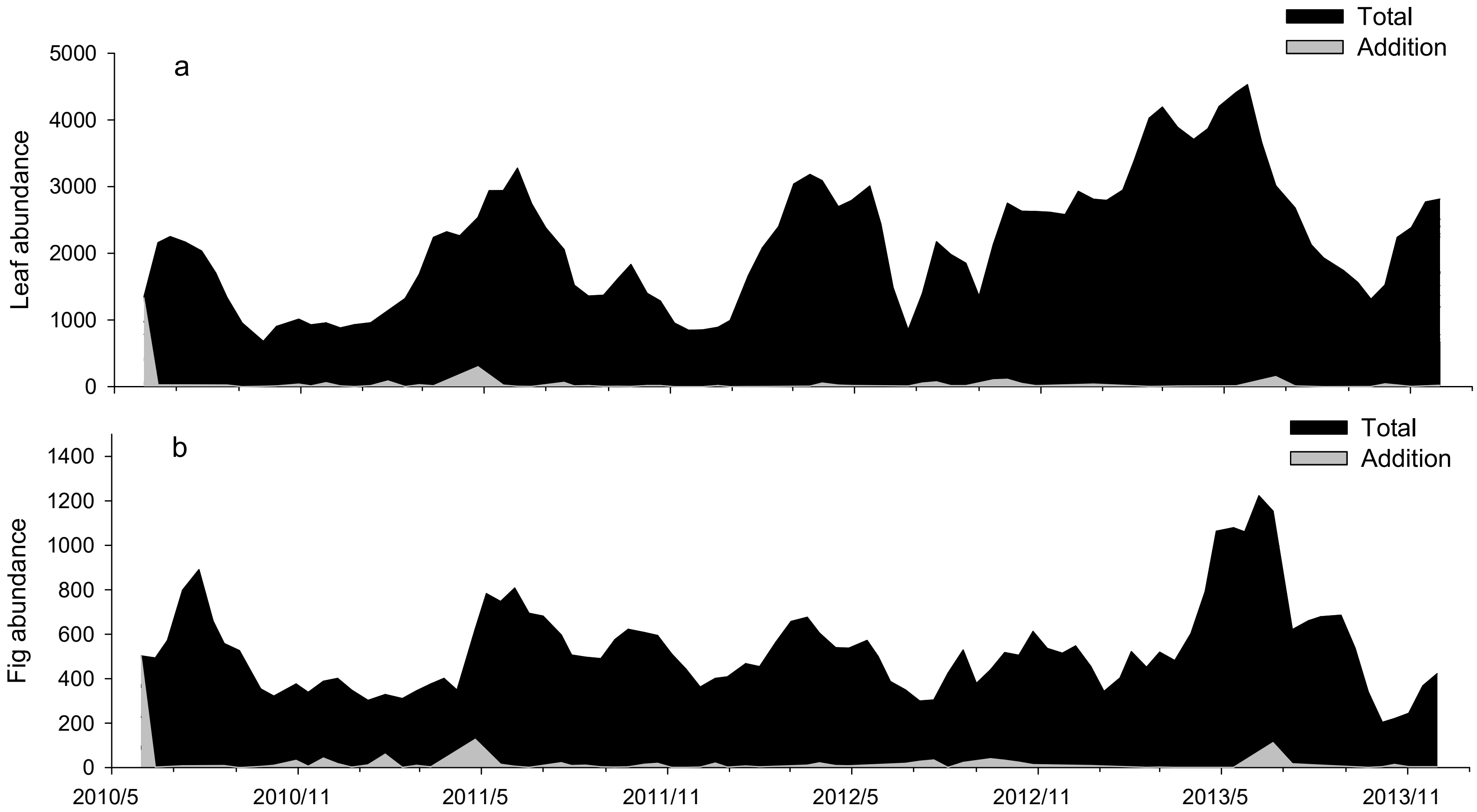

Fig. S1. The total number and additional number caused by new sample branches of leaf and fig abundance in Frog Rock Trail, a. leaf, b. fig.

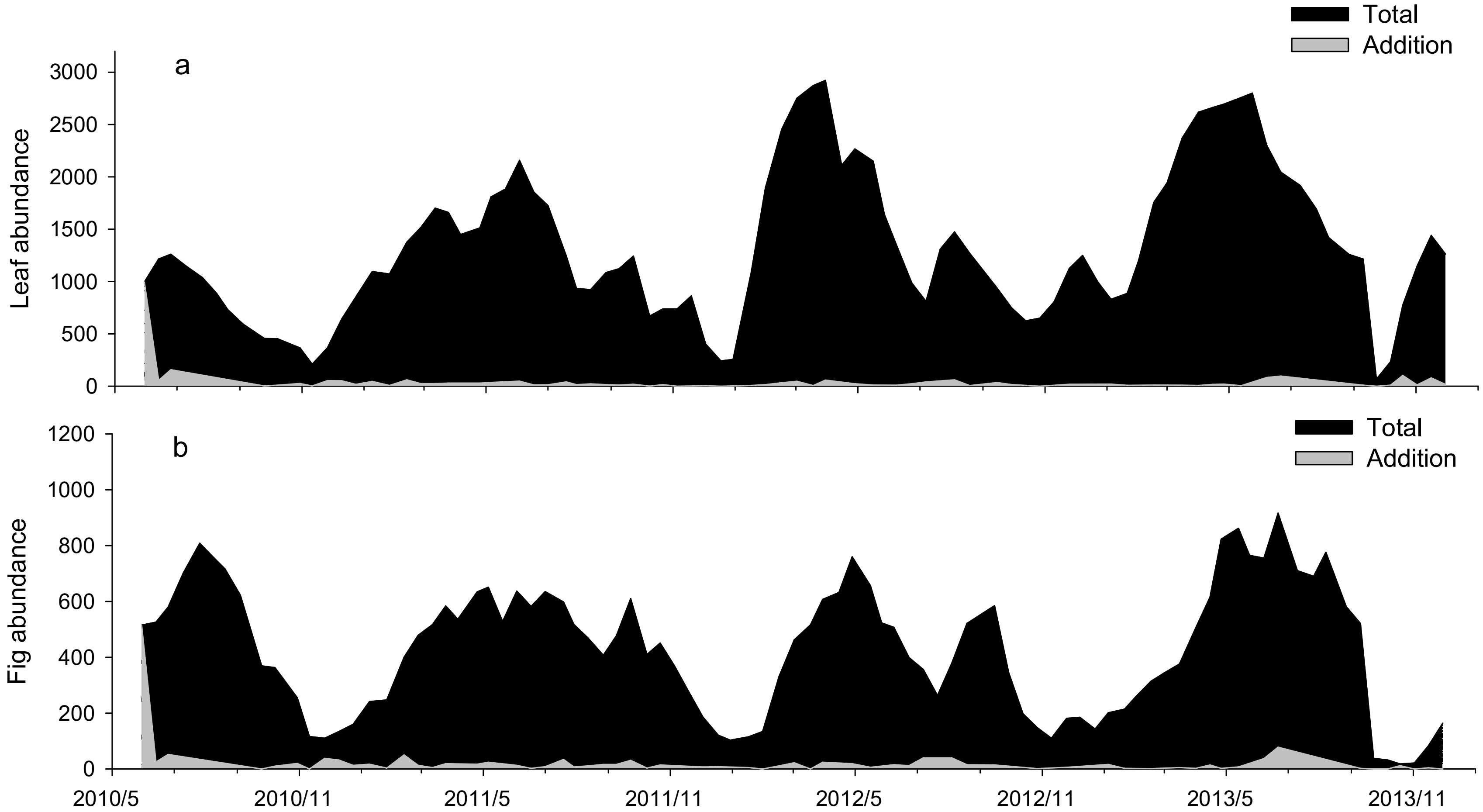

Fig. S2. The total number and additional number caused by new sample branches of leaf and fig abundance in Jialeshuei, a. leaf, b. fig.
